# Supplementary material for: Analysis of cfDNA fragmentomics metrics and commercial targeted sequencing panels
Source: Nat Commun. 2025 Oct 14;16:9122. doi: 10.1038/s41467-025-64153-z (PMC12521513; doi:10.1038/s41467-025-64153-z)
Supplement: Supplementary file 1 — Supplementary Information [file 41467_2025_64153_MOESM1_ESM.pdf]

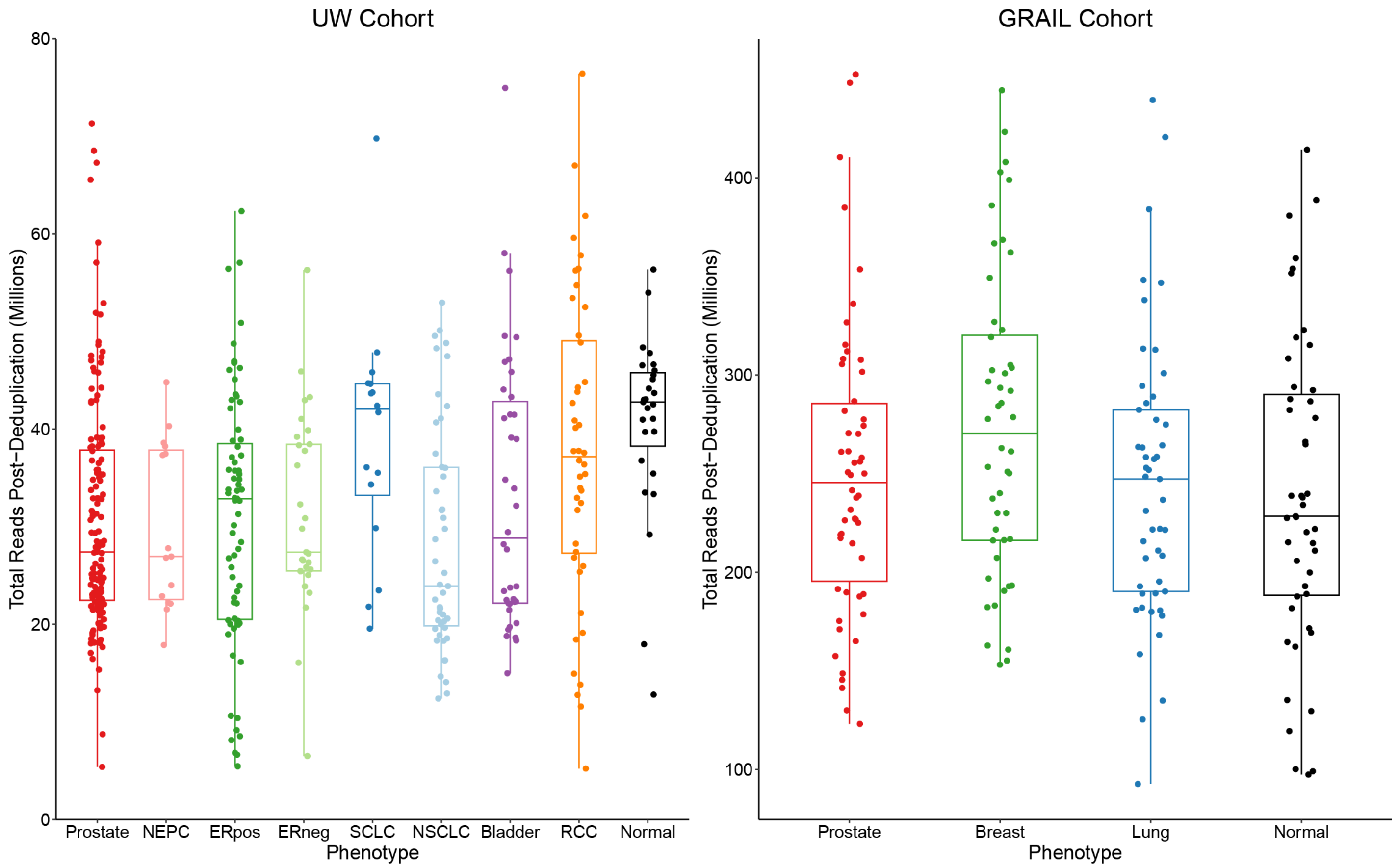

Figure S1. Total sequencing reads post-deduplication in each cohort by phenotype. NEPC, neuroendocrine prostate cancer; ERpos, ER-positive breast cancer; ERneg, ER-negative breast cancer; NSCLC, non-small cell lung carcinoma; SCLC, small cell lung carcinoma; RCC, renal cell carcinoma. Boxplots display the center as the median, with the bounds of the box as Q1 (25th percentile) and Q3 (75th percentile). Whiskers are defined by the lowest and highest value within 1.5 times the interquartile range (IQR; Q3 - Q1). Points outside of 1.5xIQR are displayed as individual points outside of the boxplot.

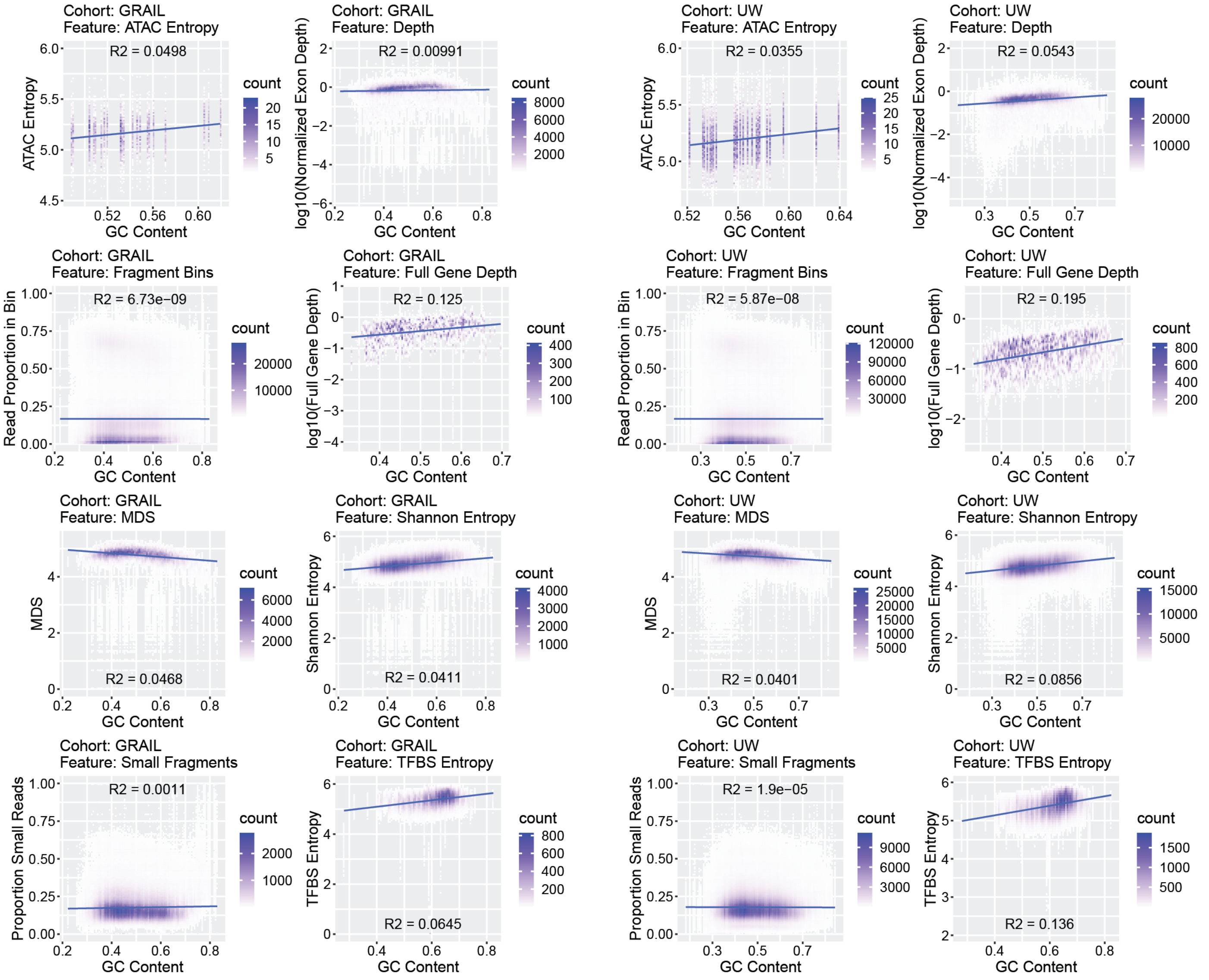

Figure S2. Correlation of GC content and fragmentomic metrics. For each genomic region tested in each feature, the GC content was calculated and plotted against the feature metric across all samples. A linear model was fitted to the data and the square of the Pearson correlation coefficient is reported (R2).

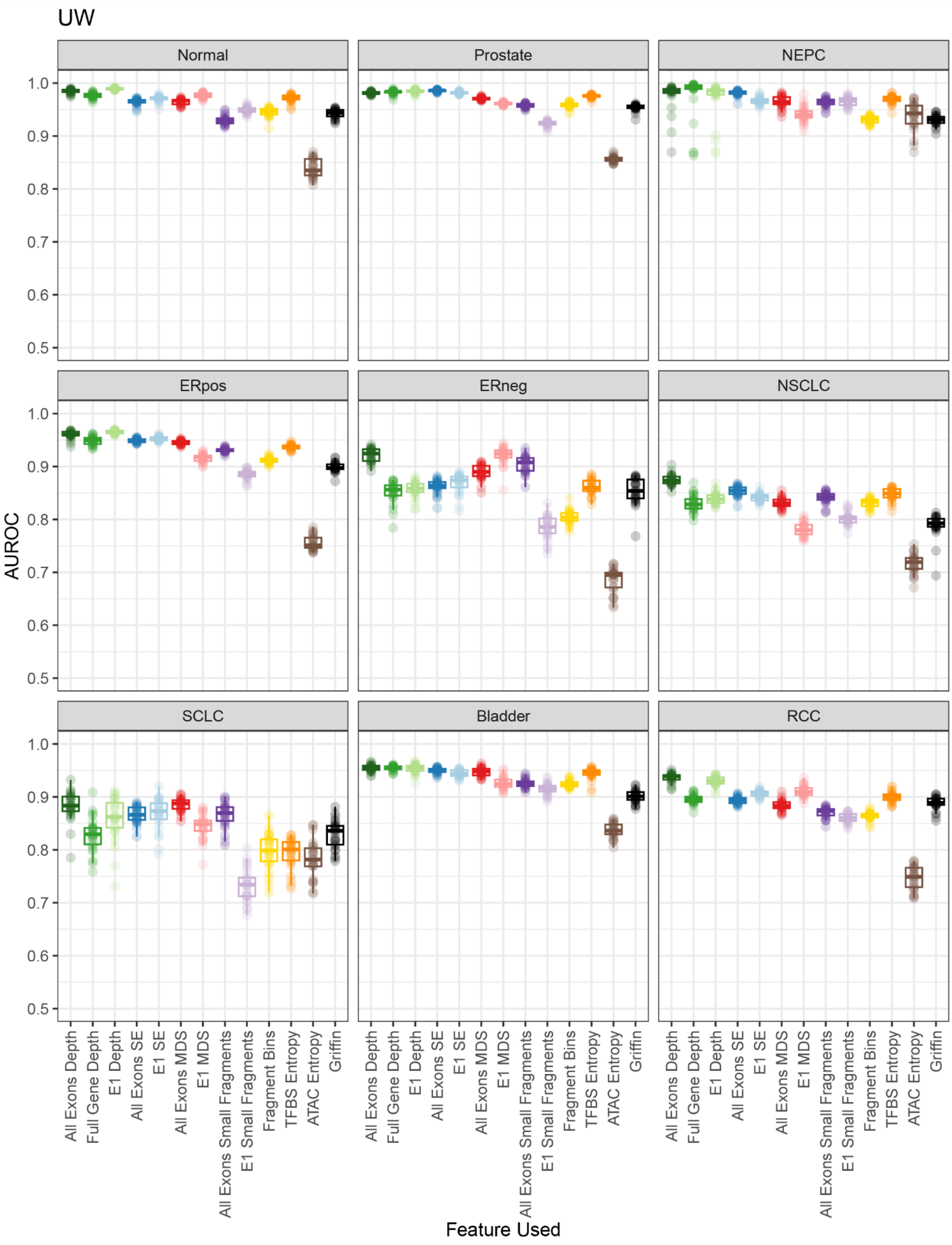

Figure S3. Overview of Fragmentomic Metric Performance in the UW Cohort including Griffin. Griffin was performed on the UW cohort as described in the report (see methods), using TFBSs from 808 TFs as the regions analyzed, and central coverage, mean coverage, and amplitude for each TF as the features for training. Boxplots display the center as the median, with the bounds of the box as Q1 (25th percentile) and Q3 (75th percentile). Whiskers are defined by the lowest and highest value within 1.5 times the interquartile range (IQR; Q3 - Q1). Points outside of 1.5xIQR are displayed as individual points outside of the boxplot.

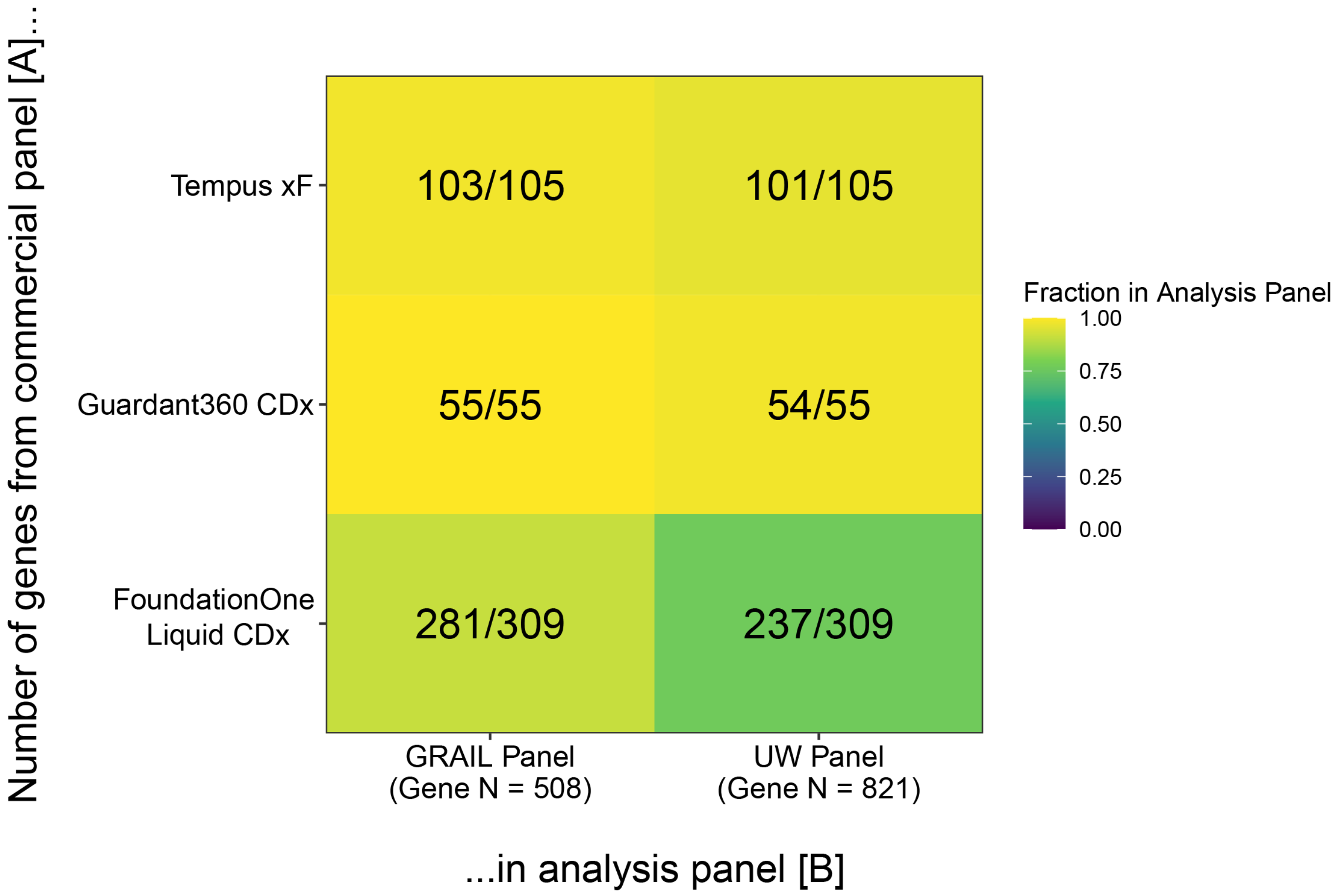

Figure S4. Overlapping Genes between Targeted Panels. The number of genes in each commercial panel which overlap with either the UW or GRAIL panel are shown as a fraction of genes in the commercial panels.

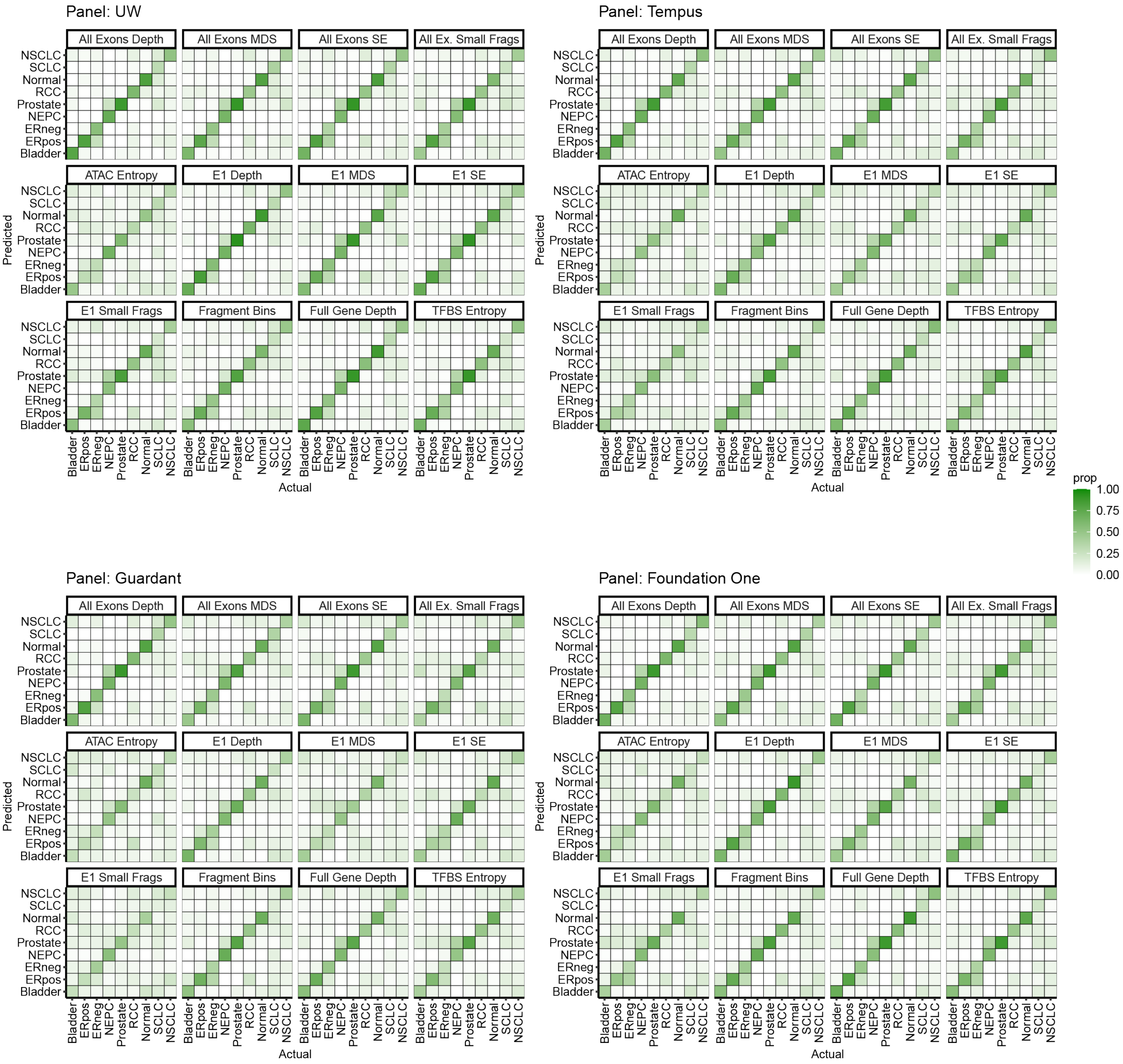

Figure S5: Confusion matrices for fragmentomics metric performance in the UW cohort. Shading represents the proportion of true (Actual) samples predicted by each model.

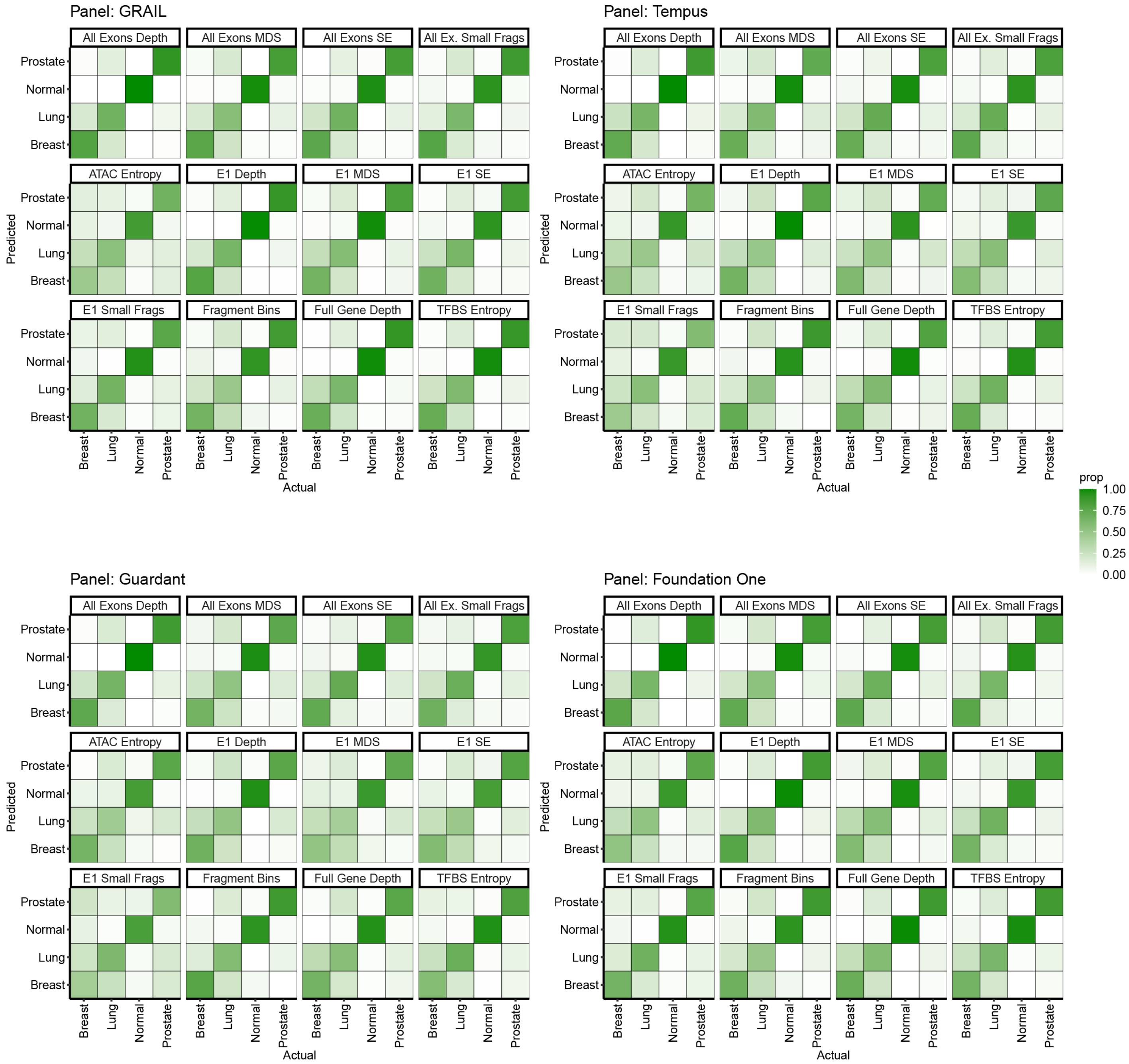

Figure S6: Confusion matrices for fragmentomics metric performance in the GRAIL cohort. Shading represents the proportion of true (Actual) samples predicted by each model.

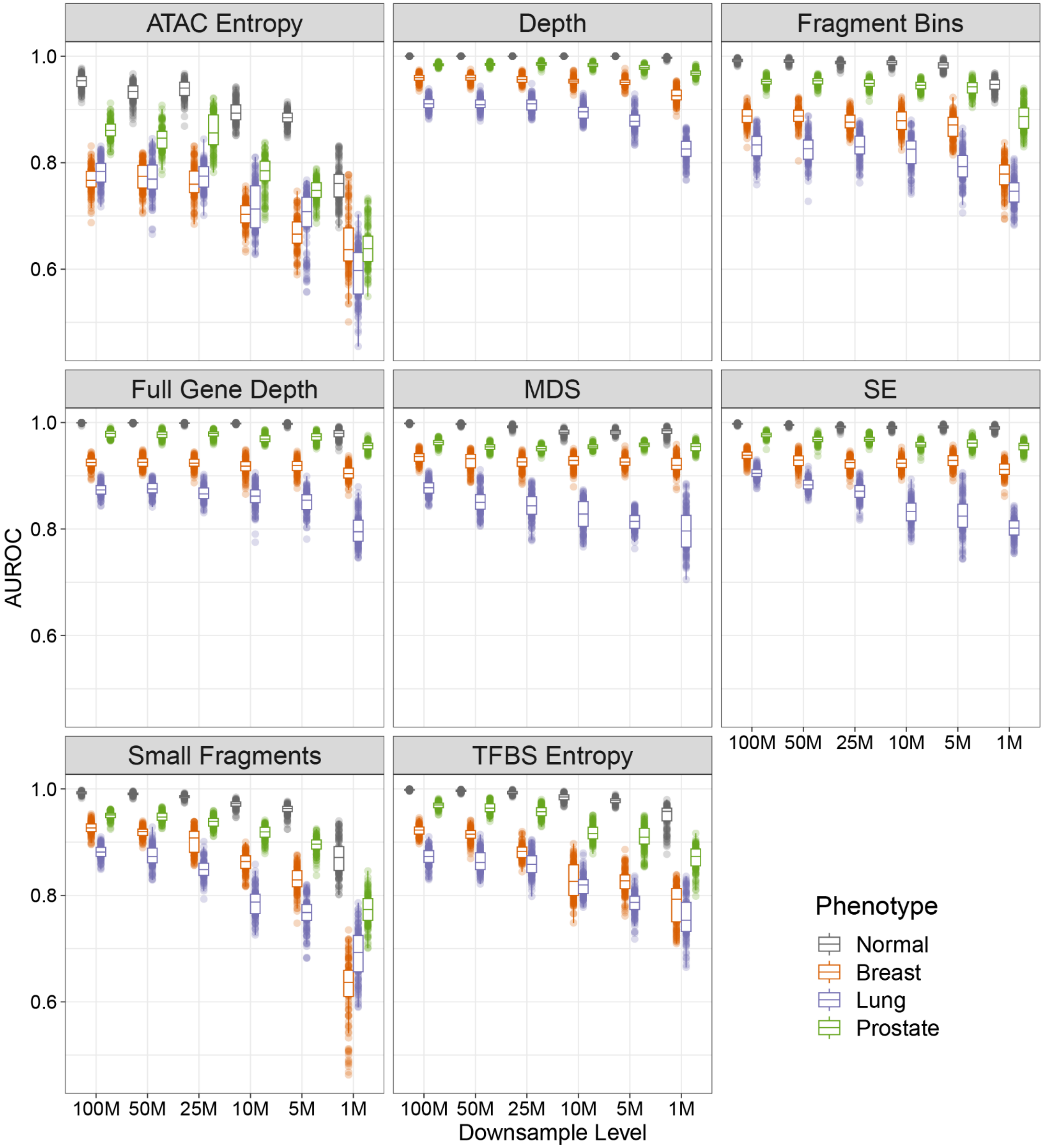

Figure S7: Down-sampling fragmentomics metric performance in the GRAIL cohort. Each sample in the GRAIL cohort was down-sampled to the level indicated (in millions of reads), and the fragmentomics metrics were calculated as previously described. These metrics were tested for their ability to predict three different cancer types along with healthy vs. cancer using a GLMnet machine learning model with 10-fold cross validation. Five independent replicates of down-sampling and twenty-five replicates of 10-fold cross validation within each down-sampling replicate were performed and boxplots of the AUROC for each metric are shown. Boxplots display the center as the median, with the bounds of the box as Q1 (25th percentile) and Q3 (75th percentile). Whiskers are defined by the lowest and highest value within 1.5 times the interquartile range (IQR; Q3 - Q1). Points outside of 1.5xIQR are displayed as individual points outside of the boxplot.

Feature Table	Gene Panel	Breast	Lung	Normal	Prostate	Mean AUROC
All Exons Depth	GRAIL	**0.958**	0.914	**1.000**	**0.986**	**0.964**
E1 Depth		0.957	0.909	**1.000**	0.977	0.961
All Exons SE		0.942	**0.915**	0.999	0.983	0.959
All Combined		0.945	0.894	**1.000**	0.983	0.956
All Exons MDS		0.945	0.893	0.999	0.968	0.951
Full Gene Depth		0.930	0.877	**1.000**	0.980	0.947
All Exons Small Fragments		0.932	0.898	0.994	0.951	0.944
TFBS Entropy		0.928	0.871	0.999	0.971	0.942
E1 SE		0.910	0.885	0.992	0.968	0.938
E1 MDS		0.904	0.833	0.999	0.951	0.922
E1 Small Fragments		0.887	0.879	0.991	0.925	0.920
Fragment Bins		0.876	0.826	0.990	0.953	0.911
ATAC Entropy		0.787	0.798	0.951	0.875	0.853
All Exons SE	FoundationOne Liquid CDx	0.947	**0.921**	0.999	**0.982**	**0.962**
All Combined		**0.954**	0.896	**1.000**	0.983	0.958
All Exons Depth		0.953	0.893	**1.000**	0.981	0.957
All Exons Small Fragments		0.936	0.914	0.993	0.963	0.952
E1 Depth		0.945	0.886	**1.000**	0.972	0.951
All Exons MDS		0.933	0.868	0.999	0.963	0.941
Full Gene Depth		0.921	0.861	0.999	0.964	0.936
E1 SE		0.907	0.874	0.987	0.963	0.933
TFBS Entropy		0.897	0.867	0.997	0.958	0.930
Fragment Bins		0.901	0.850	0.991	0.957	0.925
E1 Small Fragments		0.867	0.886	0.990	0.915	0.914
E1 MDS		0.856	0.826	0.999	0.928	0.902
ATAC Entropy		0.807	0.789	0.973	0.916	0.871
All Exons SE	Guardant360 CDx	**0.935**	**0.911**	0.995	0.957	**0.950**
All Exons Depth		0.937	0.877	**1.000**	0.967	0.945
Fragment Bins		0.918	0.898	0.993	0.961	0.943
All Combined		0.919	0.868	0.999	**0.974**	0.940
All Exons Small Fragments		0.916	0.899	0.992	0.945	0.938
TFBS Entropy		0.873	0.895	0.993	0.943	0.926
All Exons MDS		0.868	0.825	0.996	0.915	0.901
E1 Depth		0.861	0.782	0.998	0.921	0.890
ATAC Entropy		0.886	0.765	0.951	0.931	0.883
Full Gene Depth		0.853	0.780	0.994	0.896	0.881
E1 SE		0.842	0.789	0.964	0.925	0.880
E1 MDS		0.774	0.707	0.964	0.888	0.833
E1 Small Fragments		0.731	0.797	0.961	0.819	0.827
All Exons Small Fragments	Tempus xF	**0.946**	**0.919**	0.994	0.956	**0.954**
All Exons SE		0.923	0.917	0.996	0.972	0.952
All Exons Depth		0.939	0.889	**1.000**	**0.977**	0.951
All Combined		0.934	0.879	**1.000**	0.970	0.946
TFBS Entropy		0.931	0.892	0.996	0.958	0.944
Fragment Bins		0.907	0.871	0.988	0.960	0.932
All Exons MDS		0.905	0.863	0.998	0.904	0.917
Full Gene Depth		0.868	0.843	1.000	0.913	0.906
E1 Depth		0.862	0.799	1.000	0.913	0.893
E1 SE		0.828	0.811	0.981	0.920	0.885
E1 MDS		0.861	0.759	0.993	0.836	0.862
ATAC Entropy		0.786	0.745	0.971	0.885	0.846
E1 Small Fragments		0.759	0.803	0.977	0.801	0.835

Table S1. Summary table of AUROC scores for each feature and phenotype in the GRAIL cohort. AUROC value for each phenotype column represents the median AUROC of 25 replicates. The final ‘Mean AUROC’ column is the mean AUROC of the phenotypes within each feature. Bolded values represent the best AUROC for each phenotype within each gene panel.

Feature	Gene Panel	Bladder	ERneg	ERpos	NEPC	NSCLC	Normal	Prostate	RCC	SCLC	Mean AUROC
All Exons Depth	UW	0.954	**0.924**	0.960	0.985	**0.873**	0.986	0.981	**0.939**	0.884	**0.943**
E1 Depth		0.955	0.858	**0.966**	0.986	0.838	**0.989**	0.985	0.931	0.862	0.930
All Exons SE		0.951	0.864	0.950	0.983	0.854	0.966	**0.986**	0.894	0.866	0.924
E1 SE		0.944	0.874	0.951	0.966	0.842	0.972	0.981	0.908	0.873	0.924
All Combined		0.947	0.867	0.951	0.971	0.839	0.967	0.985	0.887	0.873	0.921
All Exons MDS		0.948	0.890	0.946	0.964	0.831	0.967	0.970	0.884	**0.888**	0.921
Full Gene Depth		**0.956**	0.856	0.949	**0.993**	0.828	0.977	0.984	0.896	0.829	0.919
TFBS Entropy		0.947	0.860	0.938	0.971	0.849	0.972	0.976	0.900	0.801	0.913
All Exons Small Fragments		0.925	0.908	0.931	0.965	0.843	0.929	0.957	0.873	0.870	0.911
E1 MDS		0.925	0.924	0.918	0.940	0.780	0.977	0.961	0.910	0.849	0.909
Griffin		0.901	0.854	0.897	0.931	0.793	0.944	0.954	0.889	0.836	0.889
Fragment Bins		0.922	0.803	0.911	0.932	0.833	0.947	0.959	0.864	0.799	0.886
E1 Small Fragments		0.916	0.786	0.887	0.966	0.802	0.949	0.924	0.861	0.734	0.869
ATAC Entropy		0.836	0.695	0.751	0.943	0.719	0.835	0.856	0.749	0.782	0.796
All Exons Depth	Foundation One CDx	0.939	**0.923**	**0.962**	**0.987**	**0.882**	0.983	0.979	**0.937**	0.843	**0.937**
All Combined		**0.953**	0.896	0.959	0.986	0.850	0.969	**0.984**	0.900	**0.878**	0.931
All Exons SE		0.935	0.836	0.944	0.979	0.848	0.959	0.982	0.881	0.855	0.913
Full Gene Depth		0.923	0.814	0.960	0.976	0.863	**0.991**	0.966	0.883	0.784	0.907
All Exons MDS		0.913	0.874	0.938	0.958	0.786	0.964	0.965	0.888	0.868	0.906
E1 Depth		0.934	0.824	0.945	0.961	0.818	0.986	0.957	0.903	0.800	0.903
TFBS Entropy		0.907	0.821	0.914	0.939	0.852	0.974	0.958	0.889	0.827	0.898
All Exons Small Fragments		0.894	0.875	0.911	0.960	0.828	0.926	0.945	0.848	0.834	0.891
Fragment Bins		0.910	0.832	0.916	0.947	0.817	0.959	0.963	0.889	0.731	0.885
E1 MDS		0.876	0.905	0.868	0.959	0.754	0.956	0.909	0.830	0.792	0.872
E1 SE		0.909	0.734	0.903	0.946	0.821	0.929	0.957	0.865	0.752	0.868
E1 Small Fragments		0.846	0.737	0.785	0.908	0.714	0.954	0.845	0.774	0.696	0.806
ATAC Entropy		0.809	0.728	0.804	0.846	0.714	0.896	0.873	0.752	0.698	0.791
All Exons Depth	Guardant360 CDx	**0.927**	**0.932**	**0.967**	0.983	**0.862**	0.975	**0.977**	**0.924**	**0.922**	**0.941**
All Combined		0.914	0.907	0.960	**0.985**	0.838	0.974	0.974	0.919	0.860	0.926
All Exons SE		0.921	0.808	0.883	0.943	0.804	**0.978**	0.963	0.850	0.905	0.895
All Exons MDS		0.883	0.831	0.875	0.968	0.756	0.948	0.930	0.860	0.869	0.880
Fragment Bins		0.911	0.821	0.916	0.963	0.789	0.944	0.947	0.853	0.731	0.875
Full Gene Depth		0.854	0.819	0.933	0.951	0.816	0.948	0.938	0.832	0.768	0.873
E1 Depth		0.915	0.818	0.915	0.925	0.739	0.947	0.921	0.842	0.730	0.861
TFBS Entropy		0.812	0.750	0.872	0.953	0.824	0.956	0.903	0.870	0.701	0.849
All Exons Small Fragments		0.865	0.856	0.888	0.926	0.811	0.939	0.909	0.746	0.699	0.849
E1 SE		0.832	0.734	0.830	0.965	0.761	0.920	0.923	0.786	0.707	0.829
E1 MDS		0.838	0.638	0.687	0.931	0.708	0.904	0.749	0.753	0.757	0.774
ATAC Entropy		0.742	0.672	0.751	0.821	0.727	0.909	0.832	0.753	0.695	0.767
E1 Small Fragments		0.710	0.761	0.691	0.889	0.661	0.835	0.768	0.688	0.602	0.734
All Combined	Tempus xF	**0.941**	0.896	**0.960**	0.982	**0.873**	0.970	**0.982**	**0.927**	0.865	**0.933**
All Exons Depth		0.931	**0.912**	0.955	**0.984**	0.856	**0.979**	0.972	0.918	**0.867**	0.931
All Exons SE		0.908	0.811	0.918	0.967	0.845	0.967	0.978	0.872	0.856	0.903
All Exons MDS		0.905	0.870	0.917	0.944	0.773	0.968	0.950	0.893	0.862	0.898
All Exons Small Fragments		0.909	0.895	0.898	0.956	0.836	0.938	0.935	0.840	0.778	0.887
Full Gene Depth		0.875	0.816	0.947	0.970	0.846	0.965	0.956	0.866	0.708	0.883
Fragment Bins		0.908	0.829	0.926	0.951	0.791	0.941	0.962	0.872	0.731	0.879
TFBS Entropy		0.898	0.769	0.878	0.918	0.860	0.953	0.917	0.886	0.827	0.879
E1 Depth		0.911	0.814	0.934	0.931	0.780	0.969	0.930	0.855	0.749	0.875
E1 SE		0.840	0.745	0.830	0.938	0.744	0.933	0.928	0.776	0.731	0.830
E1 MDS		0.829	0.794	0.794	0.938	0.681	0.927	0.871	0.796	0.790	0.824
E1 Small Fragments		0.813	0.792	0.745	0.910	0.723	0.881	0.798	0.718	0.605	0.776
ATAC Entropy		0.784	0.645	0.734	0.816	0.692	0.941	0.808	0.727	0.729	0.764

Table S2. Summary table of AUROC scores for each feature and phenotype in the UW cohort. AUROC value for each phenotype column represents the median AUROC of 25 replicates. The final 'Mean AUROC' column is the mean AUROC of the phenotypes within each feature. Bolded values represent the best AUROC for each phenotype within each gene panel.
