# Supplementary material for: Analysis of cfDNA fragmentomics metrics and commercial targeted sequencing panels
Source: Nat Commun. 2025 Oct 14;16:9122. doi: 10.1038/s41467-025-64153-z (PMC12521513; doi:10.1038/s41467-025-64153-z)
Supplement: Supplementary file 3 — Reporting Summary [file 41467_2025_64153_MOESM3_ESM.pdf]

nature portfolio

Corresponding author(s): Shuang George Zhao

Last updated by author(s): Aug 5, 2025

Reporting Summary

Nature Portfolio wishes to improve the reproducibility of the work that we publish. This form provides structure for consistency and transparency in reporting. For further information on Nature Portfolio policies, see our Editorial Policies and the Editorial Policy Checklist.

Statistics

For all statistical analyses, confirm that the following items are present in the figure legend, table legend, main text, or Methods section.

n/a	Confirmed	
☐	☒	The exact sample size (*n*) for each experimental group/condition, given as a discrete number and unit of measurement
☐	☒	A statement on whether measurements were taken from distinct samples or whether the same sample was measured repeatedly
☒	☐	The statistical test(s) used AND whether they are one- or two-sided<br>*Only common tests should be described solely by name; describe more complex techniques in the Methods section.*
☒	☐	A description of all covariates tested
☐	☒	A description of any assumptions or corrections, such as tests of normality and adjustment for multiple comparisons
☐	☒	A full description of the statistical parameters including central tendency (e.g. means) or other basic estimates (e.g. regression coefficient) AND variation (e.g. standard deviation) or associated estimates of uncertainty (e.g. confidence intervals)
☒	☐	For null hypothesis testing, the test statistic (e.g. *F*, *t*, *r*) with confidence intervals, effect sizes, degrees of freedom and *P* value noted<br>*Give P values as exact values whenever suitable.*
☒	☐	For Bayesian analysis, information on the choice of priors and Markov chain Monte Carlo settings
☒	☐	For hierarchical and complex designs, identification of the appropriate level for tests and full reporting of outcomes
☒	☐	Estimates of effect sizes (e.g. Cohen's *d*, Pearson's *r*), indicating how they were calculated

Our web collection on statistics for biologists contains articles on many of the points above.

Software and code

Policy information about availability of computer code

Data collection	No software was used in the collection of the data.
Data analysis	Analysis code can be found at https://github.com/Zhao-Lab-UW-DHO/fragmentomics_metrics

For manuscripts utilizing custom algorithms or software that are central to the research but not yet described in published literature, software must be made available to editors and reviewers. We strongly encourage code deposition in a community repository (e.g. GitHub). See the Nature Portfolio guidelines for submitting code & software for further information.

Data

Policy information about availability of data

All manuscripts must include a data availability statement. This statement should provide the following information, where applicable:

- Accession codes, unique identifiers, or web links for publicly available datasets
- A description of any restrictions on data availability
- For clinical datasets or third party data, please ensure that the statement adheres to our policy

Sequencing data from the GRAIL dataset is available through the European Genome Archine (ID EGAD00001005302). Pre-processed genomic start and stop coordinations (BED files) of the sequencing reads from the institutional UW cohort are uploaded to FigShare in the submission system. The institutional protocol used for the samples in this study does not allow for unrestricted access to the raw sequencing data. Requests for raw sequencing data must be submitted to the University of Wisconsin for review and approval. Data sharing requests for the samples from the two ongoing clinical trials (NCT03090165, NCT03725761) must be submitted to the trial organizers for approval.

Research involving human participants, their data, or biological material

Policy information about studies with human participants or human data. See also policy information about sex, gender (identity/presentation), and sexual orientation and race, ethnicity and racism.

Reporting on sex and gender	Sex/gender data was not utilized in the analysis of this data. Genes on the Y chromosome were specifically excluded from the fragmentomics models.
Reporting on race, ethnicity, or other socially relevant groupings	Race, ethnicity, or other socially relevant grouping were not utilized in the analysis of this data.
Population characteristics	The University of Wisconsin (UW) cohort comprises metastatic cancer patients enrolled in a liquid biology collection protocol which has been approved by an institutional review board (2014-1214) in addition to samples from two ongoing clinical trials (NCT03090165, NCT03725761). Healthy blood samples were purchased from the University of Wisconsin Carbone Cancer Center (UWCCC) tissue BioBank and processed in the same manner as the cancer samples.
Recruitment	Patients were recruited using the above protocols.
Ethics oversight	University of Wisconsin - Madison IRB

Note that full information on the approval of the study protocol must also be provided in the manuscript.

Field-specific reporting

Please select the one below that is the best fit for your research. If you are not sure, read the appropriate sections before making your selection.

- [x] Life sciences
- [ ] Behavioural & social sciences
- [ ] Ecological, evolutionary & environmental sciences

For a reference copy of the document with all sections, see nature.com/documents/nr-reporting-summary-flat.pdf

Life sciences study design

All studies must disclose on these points even when the disclosure is negative.

Sample size	431 cfDNA samples were collected at the University of Wisconsin - Madison (38 bladder cancer, 71 ER-positive breast cancer, 29 ER-negative breast cancer, 15 neuroendocrine prostate cancer (NEPC), 144 prostate adenocarcinoma, 44 renal cell carcinoma (RCC), 16 small cell lung cancer (SCLC), 47 non-small cell lung cancer (NSCLC), and 27 healthy i.e. non-cancer donors)
Data exclusions	Cancer types with an insufficient number of samples (i.e. <= 5) were excluded from this study.
Replication	NA. There was only one sample per patient
Randomization	For machine learning analysis, samples were randomly assigned into either training or validation cohorts for analysis.
Blinding	Blinding is not relevant for this study, as knowledge of the cancer type metadata is required to randomly split data into training and validation cohorts as well as to assess performance of the machine learning model.

Reporting for specific materials, systems and methods

We require information from authors about some types of materials, experimental systems and methods used in many studies. Here, indicate whether each material, system or method listed is relevant to your study. If you are not sure if a list item applies to your research, read the appropriate section before selecting a response.

Materials & experimental systems

n/a	Involved in the study	
x		Antibodies
x		Eukaryotic cell lines
x		Palaeontology and archaeology
x		Animals and other organisms
x		Clinical data
x		Dual use research of concern
x		Plants

Methods

n/a	Involved in the study	
x		ChIP-seq
x		Flow cytometry
x		MRI-based neuroimaging

Plants

Seed stocks	NA
Novel plant genotypes	NA
Authentication	NA
